# Supplementary figures and images for: Differential Transcriptomic Features of Peripheral Blood Mononuclear Cells in Pulmonary Sarcoidosis with and Without Extrapulmonary Lesions in an East Asian Population
Source: Biomedicines. 2025 Dec 7;13(12):2998. doi: 10.3390/biomedicines13122998 (PMC12731017; doi:10.3390/biomedicines13122998)

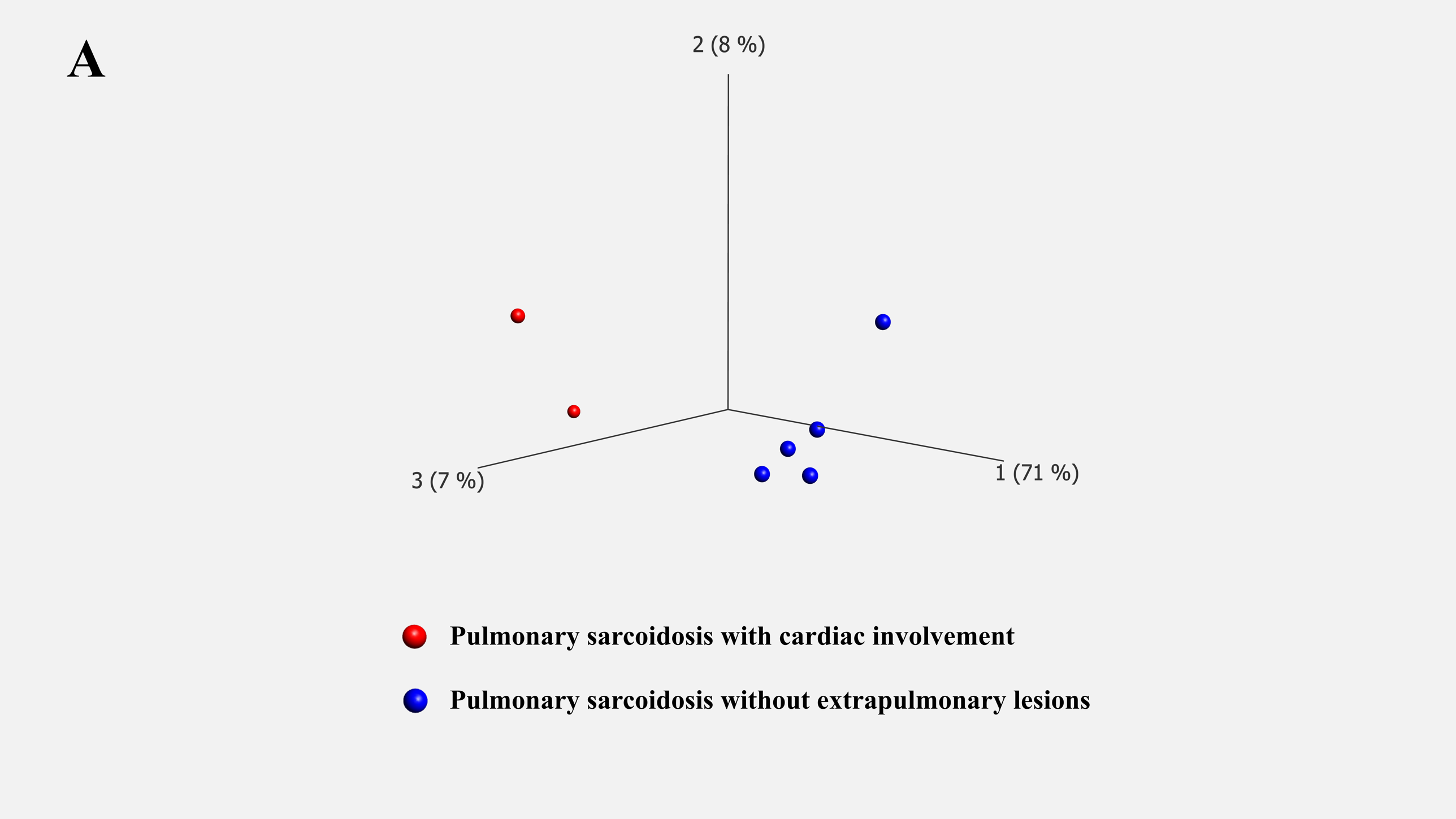

Supplement: Supplementary file 1 [file biomedicines-13-02998-s001.zip › Supplementary_Figure_S1A.TIF]

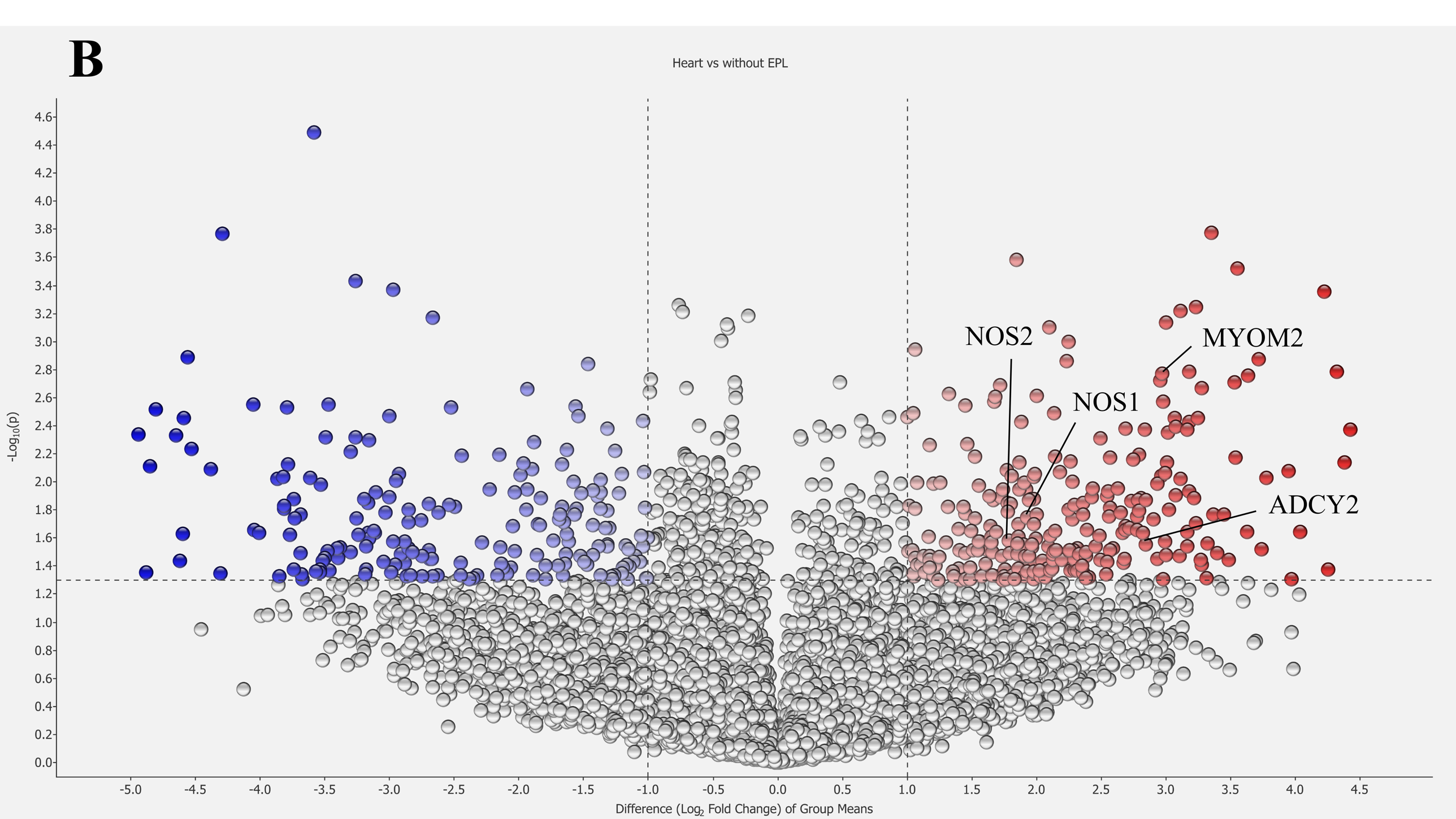

Supplement: Supplementary file 1 [file biomedicines-13-02998-s001.zip › Supplementary_Figure_S1B.tif]

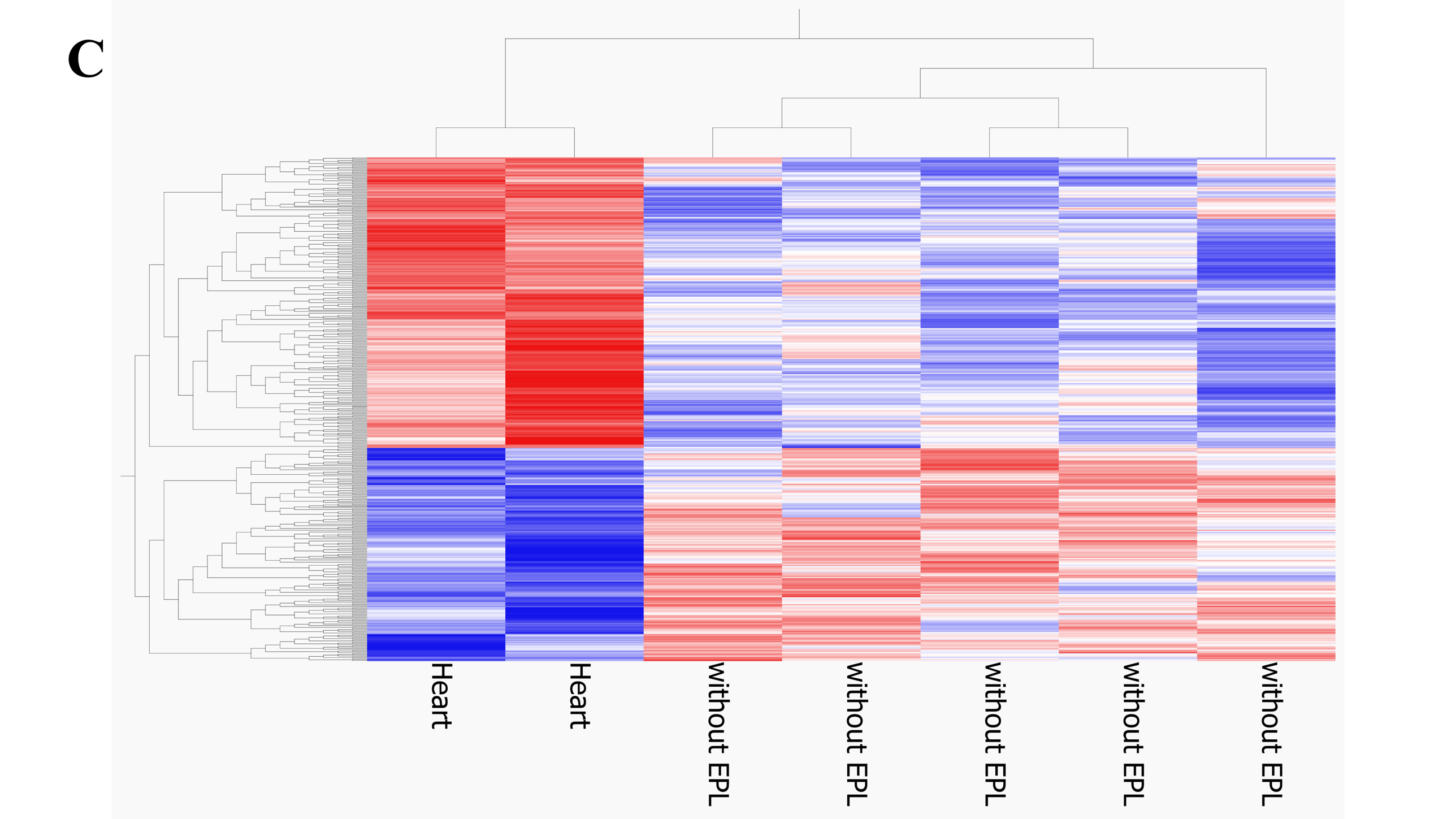

Supplement: Supplementary file 1 [file biomedicines-13-02998-s001.zip › Supplementary_Figure_S1C.TIF]
